# Predictors of developmental surveillance completion at six months of age in south western Sydney

**DOI:** 10.1111/cch.12425

**Published:** 2016-12-01

**Authors:** B. J. Overs, S. Woolfenden, K. Williams, B. Jalaludin, E. L. Axelsson, C. Dissanayake, J. Descallar, S. Harvey, D. Beasley, E. Murphy, V. Eapen, the ‘Watch Me Grow’ Study Group

**Affiliations:** ^1^ University of New South WalesSydneyNSWAustralia; ^2^ Sydney Children's Hospitals NetworkSydneyNSWAustralia; ^3^ Royal Children's HospitalParkvilleVICAustralia; ^4^ Murdoch Children's Research InstituteParkvilleVICAustralia; ^5^ University of MelbourneMelbourneVICAustralia; ^6^ South Western Sydney Local Health DistrictLiverpoolNSWAustralia; ^7^ Ingham Institute for Applied Medical ResearchLiverpoolNSWAustralia; ^8^ Australian National UniversityCanberraACTAustralia; ^9^ Academic Unit of Child PsychiatrySouth Western Sydney Local Health District LiverpoolNSWAustralia; ^10^ Olga Tennison Autism Research CentreLa Trobe UniversityMelbourneVICAustralia; ^11^ South Western Sydney Clinical SchoolUniversity of New South WalesLiverpoolNSWAustralia; ^12^ Child and Family HealthNew South Wales Ministry of HealthSydneyNSWAustralia

**Keywords:** cultural and linguistic diversity, developmental surveillance, Parents' Evaluation of Developmental Status, socioeconomic disadvantage

## Abstract

**Background:**

While developmental surveillance programs promote early identification of child developmental problems, evidence has indicated suboptimal uptake. This study aimed to identify predictors of developmental surveillance completion at 6 months postpartum.

**Methods:**

Questionnaires were administered to the parents of 510 infants who were born in south western Sydney, Australia over a 22‐month period. Attendance for developmental screening and completion of the *Parents' Evaluation of Developmental Status (PEDS)* at 6 months postpartum were modelled separately using multivariable logistic regression.

**Results:**

Developmental surveillance attendance was predicted by higher levels of maternal education, annual income and being informed about checks. PEDS completion at 6 months of age was predicted by higher income and being informed, as well as being married, employed, speaking English at home, full‐term birth and the professional status of the practitioner completing the check.

**Conclusions:**

Barriers to developmental surveillance included low socioeconomic status, linguistic diversity and possible gaps in parental knowledge and professional education. Developmental surveillance rates may be increased by the addition of targeted parental and professional support within current universal frameworks.

## Background

Child developmental problems represent an ever increasing health and economic concern (Jarbrink *et al.* 2003; Matson & Kozlowski 2011; Lavelle *et al.* 2014). While early intervention may reduce the impact of developmental disorders (McConachie & Diggle 2007; Rogers & Vismara 2008; Howlin *et al.* 2009; Einfeld *et al.* 2013), timely identification of children who could benefit remains a significant challenge (Chung *et al.* 2006; Eapen *et al.* 2014).

Developmental surveillance (DS) is a means of identifying children at risk of developmental problems and promoting early diagnosis (Committee on Children With Disabilities 2001; National Health and Medical Research Council 2002). In New South Wales (NSW), regular child health screening commences at 1–4 weeks postpartum (Ministry of Health‚ New South Wales 2013; Eapen *et al.* 2014), and is usually provided by a Child and Family Health Nurse or general practitioner (GP). The DS schedule is contained in the child's Personal Health Record (PHR), given to parents at the birth of their child, and verbal information about DS is provided opportunistically during the perinatal period.

The *Parents' Evaluation of Developmental Status* (PEDS) is included in the PHR at each surveillance ‘check’ from 6 months to 4 years of age, and is also available in multiple languages online (Glascoe 1999; Ministry of Health‚ New South Wales). This brief 10‐question screening tool aids detection of developmental problems by exploring parents' concerns about their child's development (Glascoe 2006; Schonwald *et al.* 2009). It should be completed by parents prior to each scheduled ‘check’ or otherwise the questions should be asked during the check and filled out in the PHR.

Despite the availability of DS programs, their uptake is suboptimal. The 2009–2010 report on child health from the Centre for Epidemiology and Research in NSW (2008) found that 50% of children aged less than 12 months had attended an early childhood health centre, and only 15% of non‐attendees reported use of another services. Between 1 and 4 years of age, attendance decreased to 33%, with similar rates observed internationally (Chung *et al.* 2006).

While previous research has identified numerous barriers to universal healthcare uptake, little is known about the barriers specific to DS. Risk factors for reduced universal healthcare uptake include low socioeconomic status (SES)(Comino & Harris 2003; Fort Harris *et al.* 2004), and cultural and linguistic diversity (Carbone *et al.* 2004; Fort Harris, Harris, & Roland; Murray & Skull 2005; Schyve 2007; Woolfenden *et al.* 2014), which represent key potential barriers to DS in ethnically diverse and economically disadvantaged regions like south western Sydney (Sydney South West Area Health Service 2005).

The present study aimed to identify predictors of non‐attendance at 6‐month DS, and predictors for non‐completion of the PEDS, using the Andersen Behavioural Model of Health Service Use as a framework (Andersen 1995; Andersen & Davidson 2007). This model has been extensively applied in studies of health service use (Babitsch *et al.* 2012; Woolfenden *et al.* 2014).

## Methods

### Participants and ethical approval

Participants comprised the parents of 510 infants born in south western Sydney between October 2011 and July 2013. These were a subsample of a birth cohort recruited for the ‘Watch Me Grow’ study, a mixed methods study of developmental risk and surveillance in NSW. Recruitment methods have been detailed elsewhere (Eapen *et al.* 2014; Woolfenden *et al.* 2016). Participants who had completed the first two study components (baseline and 6‐month follow‐up) at the time of analysis were selected for inclusion. Retention at 6 months was 75%, with 3% declining to continue and 22% unreachable by telephone. While largely representative of the culturally diverse and economically disadvantaged population of south western Sydney, the ‘Watch Me Grow’ cohort demonstrated higher instances of specific risk factors for developmental problems (Woolfenden *et al.* 2016), making this sample ideal for the examination of risk factors for DS uptake. Ethical approval was granted by the Human Research Ethics Committees of the University of New South Wales and South Western Sydney Local Health District.

### Measurement tools

Sociodemographic and health service use information was obtained using a baseline questionnaire at birth and a follow‐up telephone interview at 7 to 12 months postpartum. Both questionnaires were developed in collaboration with local healthcare providers, and incorporated questions from other Australian birth cohort studies (Nicholson & Sanson 2003; Comino *et al.* 2010; The SEARCH Investigators 2010). For participants who could not complete the baseline questionnaire during recruitment, it was completed retrospectively during the follow‐up interview (*n* = 77). As part of this interview, the 6‐month PEDS was completed with the parent if answers had not been recorded previously in the PHR.

### Statistical analyses

All analyses were conducted in SPSS version 22. Group differences were examined using the chi‐square and non‐parametric Mann–Whitney *U* tests (because of non‐normal distribution).

Single predictor binary logistic regression models were used to investigate sociodemographic and service use characteristics associated with two separate dependant variables: attendance of the 6‐month DS and completion of 6‐month PEDS in the PHR for those who attended a well child check. All independent variables were selected from one of the two study questionnaires on the basis of previous research highlighting their value in predicting healthcare uptake. These factors were semantically categorised according to the Andersen model (Tables 1 and 2). Multi‐categorical variables were dichotomised based on group differences from previous research, or socially and economically relevant groupings. Assuming equal sized groups, we had at least 80% power (with alpha of .05) to detect differences in prevalence between 10 and 15%. For each dependent variable, any factor with *p* < .25 in a single predictor logistic regression model was included in a multivariable logistic regression model. The final model was determined using the backward selection method, and discriminatory power was measured by the coefficient of discrimination statistic (Tjur 2009).

**Table 1:** Attendance rates at 6‐month developmental screening and completion of 6‐month Parents' Evaluation of Developmental Status (for those who attended screening) by Andersen Model Factor and categorical group

					Six‐month Developmental Surveillance attendance			Six‐month *Parents*' *Evaluation of Developmental Status* completion		
Andersen Model Factor	Characteristic		*N*	Missing	*n*	%	*p*	*n*	%	*p*
**Predisposing characteristics**										
	Gender (child)	Male	239	0	205	86	.92	91	48	.27
		Female	271		230	86		116	54	
	Marital status (mother)	Married	406	0	355	88	<.01	164	50	.29
		Not married	104		80	78		43	57	
	Have second parent (child)	Yes	480	3	412	86	.18	199	52	.06
		No	27		20	77		5	28	
	Country of birth (mother)	Australia	224	3	192	86	.90	109	61	<.01
		Other	283		240	86		97	44	
	Country of birth (father)	Australia	208	23	177	85	.57	101	61	<.01
		Other	278		239	87		99	45	
	Education level (mother)	Tertiary	332	5	297	90	<.01	146	53	.23
		Below Tertiary	173		133	78		59	47	
	Education level (father)	Tertiary	309	30	271	88	.19	129	51	.73
		Below Tertiary	170		142	84		70	53	
	Employment status (mother)	Employed	323	4	288	89	<.01	156	58	<.01
		Unemployed	183		144	80		50	37	
	Employment status (father)	Employed	483	26	373	86	.84	185	53	.15
		Unemployed	27		41	87		15	41	
	Number of children in family	Single	297	5	241	82	<.01	106	48	.10
		Multiple	208		189	91		99	56	
	English spoken at home	Yes	345	1	297	87	.40	155	56	<.01
		Never	164		137	84		51	41	
**Enabling resources**										
	Annual income	≥$25 000	396	45	351	89	<.01	178	54	<.01
		<$25 000	69		46	68		12	29	
	Income covers costs	Yes	461	13	398	87	.04	197	53	.02
		No	36		26	74		7	28	
	Six‐month immunisation complete	Yes	487	4	416	86	.38	200	52	.06
		No	19		15	79		3	23	
	Perceived support	Get enough	433	21	364	85	.22	179	33	.01
		Don't get enough	56		51	91		16	53	
	Informed about developmental surveillance	Yes	235	32	212	90	<.01	120	61	<.01
		No	243		195	82		77	42	
	Practitioner who completed 6‐month screening	Child and Family Health Nurse	121	0	—	—	—	94	84	<.01
		Other	314		—	—		113	39	
**Need**										
	Physical health problems in first 6 months (child)	Yes	103	7	90	87	.60	46	54	.45
		No	400		338	85		156	49	
	Health problems pre pregnancy (mother)	Yes	68	0	62	91	.19	36	64	.04
		No	442		373	85		171	49	
	Health problems during pregnancy (mother)	Yes	136	1	112	84	.36	59	57	.16
		No	373		322	87		148	49	
	Premature birth (child)	Full‐term	473	0	402	85	.16	193	52	.30
		Premature	37		33	94		14	42	
	Family history of psychosocial problems	Yes	81	5	70	86	.87	40	61	.09
		No	424		360	86		164	49	
	Smoked during pregnancy (mother)	Yes	43	4	34	79	.19	16	50	.90
		No	463		397	86		189	51	
	Alcohol consumption during pregnancy (mother)	Yes	22	4	19	86	.94	10	59	.52
		No	484		412	86		195	51	
	Breastfed after birth	Yes	455	0	394	87	.03	186	51	.72
		No	55		41	76		21	54	

**Table 2:** Attendance rates at 6‐month developmental screening and completion of 6‐month Parents' Evaluation of Developmental Status (for those who attended screening) by Andersen Model Factor for continuous predictors

					*P* values	
Andersen Model Factor	Characteristic	Missing	Median	Range	Six‐month Developmental Surveillance attendance	Six‐month *Parents*' *Evaluation of Developmental Status* completion
**Predisposing characteristics**						
	Mother's age (years)	3	30.0	13 – 46	.10	.64
	Father's age (years)	28	33.5	16 – 67	.42	.07
	Number of sources for child development information	4	3.0	1 – 14	<.01	<.01
**Need**						
	Number of *Parents' Evaluation of Developmental Status* concerns	28	0.0	0 – 8	.83	<.01
	Child birth weight (g)	1	3335.0	838 – 4880	.19	.85

## Results

### Sample characteristics

Tables 1 and 2 provide the distribution and simple logistic regression results for all factors. The majority of parents were married, born overseas, educated above secondary level and employed at baseline. English was never spoken at home for 32% of the sample, and 15% reported an annual household income below the poverty line (<$25 000). DS checks were completed primarily by a GP (65%), a Child and Family Health Nurse (28%) or a paediatrician (4%). Only 46% of parents reported being told about DS checks.

### Attendance for 6‐month Developmental Surveillance

**Figure 1:**
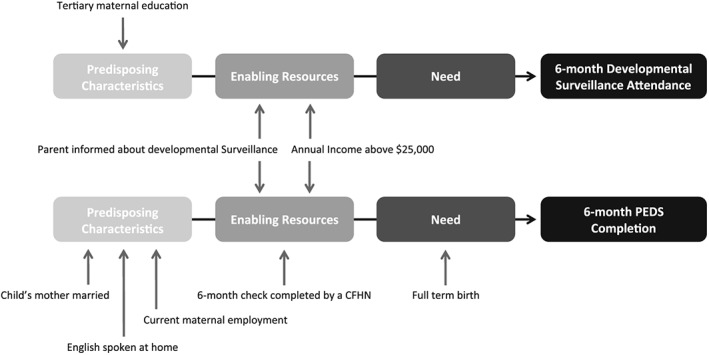
Andersen Behavioural Models of 6‐month developmental surveillance attendance and Parents' Evaluation of Developmental Status completion, with predictors based on final multivariable logistic regression models.

For the total sample, the 6‐month DS attendance rate was 85% (Table 1). The final regression model included three significant predictors of attendance (Table 3), representing one predisposing characteristic and two enabling resources (Fig. 1). Increased odds of DS attendance were observed for mothers with a tertiary education (OR = 2.09, *p* = .02), families with an annual income over $25 000 (OR = 2.55, *p* = .02) and parents who were informed about DS (OR = 2.22, *p* = .01). The estimated coefficient of discrimination was *D* = .08 (Tjur 2009), suggesting low explanatory power.

**Table 3:** Final multivariable logistic regression models

Model	Predictor	OR	95% CI
Six‐month Developmental Surveillance attendance	Maternal education (tertiary and above vs. below tertiary)	2.09	1.11 – 3.94
	Annual income ($25 000 and above vs. below $25 000)	2.56	1.15 – 5.66
	Informed about developmental surveillance (informed vs. uninformed)	2.22	1.18 – 4.2
Six‐month Parents' Evaluation of Developmental Status completion	Marital status (married vs. unmarried)	2.17	1.02 – 4.64
	Maternal employment (employed vs. unemployed)	3.09	1.71 – 5.61
	English spoken at home (English vs. no English)	2.06	1.13 – 3.77
	Full‐term birth (full‐term vs. preterm)	2.87	1.11 – 7.37
	Professional who complete 6‐month check (Child and Family Health Nurse vs. other)	8.12	4.04 – 16.34
	Annual income ($25 000 and above vs. below $25 000)	3.38	1.13 – 10.05
	Informed about developmental surveillance (informed vs. uninformed)	1.96	1.15 – 3.33

### Completion of PEDS in the Personal Health Record

Amongst 6‐month DS attendees, 51% reported completion of the PEDS in the PHR (Table 1), with an additional 7% reporting that the PEDS questions were asked by a health practitioner but not recorded in the PHR. Seven factors were associated with increased PEDS completion in the final regression model (Table 3): mothers who were unmarried (OR = 2.17, *p* = .05) or unemployed (OR = 3.09, *p* < .01), English spoken at home (OR = 2.06, *p* = .02), annual income over $25 000 (OR = 3.38, *p* = .03), being informed of DS (OR = 1.96, *p* = .01), full‐term birth (OR = 2.87, *p* = .03) and Child and Family Health Nurse completing DS (OR = 8.12, *p* < .01). Within the Anderson Behavioural Model, these predictors represented three predisposing characteristics, one enabling resource and one need factor (Fig. 1). The estimated coefficient of discrimination was D = .28 (Tjur 2009), indicating low explanatory power.

## Discussion

The rate of self‐reported DS attendance for a 6‐month check (85%) was more promising than the 65% reported by the Centre for Epidemiology and Research (2008); however, there was disparity between DS attendance and PEDS completion at 6 months (51%). It is possible that a separate PEDS form was used in some cases, as 7% of parents recalled the PEDS questions being asked when it was not filled out in the PHR. However, the issue of the remaining 42% of attendees with uncompleted PEDS requires further examination.

Predictors of increased DS attendance and PEDS completion included multiple factors previously identified as providing protection from child developmental problems (Najman *et al.* 1992; To *et al.* 2004). Families with an annual income over $25 000 were more likely to attend DS and have the PEDS recorded in the PHR, supporting previous reports of less universal healthcare service use amongst those of low SES (Fort Harris *et al.* 2004). In accordance with Comino and Harris (2003), tertiary maternal education predicted DS attendance, while significantly more PEDS forms were completed for employed mothers and children born at term. As low SES and preterm birth are well‐established predictors of developmental problems (Najman *et al.* 1992), these patterns of attendance and PEDS completion indicate the presence of the inverse care law (Tudor Hart 1971) with those at greatest developmental risk not accessing or receiving the recommended care. These findings demonstrate the importance of targeted solutions to support DS within these vulnerable populations.

Another potential target for improving DS uptake is parental awareness. While only 46% of parents reported being told about DS, this factor significantly increased attendance by 12% and PEDS completion by 19%. Increased information about DS and regular reminders of upcoming checks may provide a simple means of bolstering uptake.

While the strongest predictor of PEDS completion was having a Child and Family Health Nurse conduct the 6‐month developmental screen, the majority of DS (65%) was conducted by GPs. PEDS completion may therefore be increased by improving parental awareness of the Child and Family Health Nurse's role, and increasing access to standardised training in DS administration by all primary health professionals. This is supported by the work of Woolfenden *et al.* (2015) who found that one third of a sample of 277 NSW GPs and practice nurses felt that their training in childhood development was poor, and 90% desired further training.

The increased PEDS completion in English‐speaking households adds to the body of research suggesting cultural and linguistic barriers to universal healthcare uptake (Carbone *et al.* 2004; Fort Harris, Harris, & Roland; Woolfenden *et al.* 2014), and is pertinent to ethnically diverse areas. Expanding upon the methods of disseminating PHR translations may increase DS access within CALD populations.

### Limitations and strengths

The possibility of recall bias for questionnaires completed retrospectively is a potential limitation (15%). Retention at 6 months was 75%, and it is possible that DS was higher in this sample because of factors associated with continued research engagement. Strengths of this research included sample diversity and the inclusion of low SES families who were broadly representative of the population of south western Sydney.

## Conclusion

Recent advances have highlighted the benefit of programs aimed at early identification of children at developmental risk (Oberklaid *et al.* 2013). However, our findings indicate barriers to implementation, particularly within low SES and linguistically diverse populations. This research provides an argument and policy focus for universal access to programs promoting healthy child development along with targeted support commensurate with additional needs.

**Key messages**

- Child developmental problems present a significant health and economic concern.
- Developmental surveillance programs aim to identify children at risk of developmental problems and lead to early and accurate diagnosis.
- Socioeconomic disadvantage, linguistic barriers and gaps in parental knowledge and professional education contribute to suboptimal uptake of developmental surveillance.
- A policy focus on the provision of targeted support within a universal framework may facilitate timely identification of children who could benefit from early intervention.
